# Analysis of Neurotrophic Factors in Limb and Extraocular Muscles of Mouse Model of Amyotrophic Lateral Sclerosis

**DOI:** 10.1371/journal.pone.0109833

**Published:** 2014-10-15

**Authors:** Vahid M. Harandi, Susanne Lindquist, Shrikant Shantilal Kolan, Thomas Brännström, Jing-Xia Liu

**Affiliations:** 1 Department of Integrative Medical Biology, Section for Anatomy, Umeå University, Umeå, Sweden; 2 Department of Clinical Sciences, Pediatrics, Umeå University, Umeå, Sweden; 3 Department of Medical Biosciences, Pathology, Umeå University, Umeå, Sweden; Inserm, France

## Abstract

Amyotrophic lateral sclerosis (ALS) is currently an incurable fatal motor neuron syndrome characterized by progressive weakness, muscle wasting and death ensuing 3–5 years after diagnosis. Neurotrophic factors (NTFs) are known to be important in both nervous system development and maintenance. However, the attempt to translate the potential of NTFs into the therapeutic options remains limited despite substantial number of approaches, which have been tested clinically. Using quantitative RT-PCR (qRT-PCR) technique, the present study investigated mRNA expression of four different NTFs: brain-derived neurotrophic factor (BDNF), neurotrophin-3 (NT-3), neurotrophin-4/5 (NT-4) and glial cell line-derived neurotrophic factor (GDNF) in limb muscles and extraocular muscles (EOMs) from SOD1^G93A^ transgenic mice at early and terminal stages of ALS. General morphological examination revealed that muscle fibres were well preserved in both limb muscles and EOMs in early stage ALS mice. However, in terminal ALS mice, most muscle fibres were either atrophied or hypertrophied in limb muscles but unaffected in EOMs. qRT-PCR analysis showed that in early stage ALS mice, NT-4 was significantly down-regulated in limb muscles whereas NT-3 and GDNF were markedly up-regulated in EOMs. In terminal ALS mice, only GDNF was significantly up-regulated in limb muscles. We concluded that the early down-regulation of NT-4 in limb muscles is closely associated with muscle dystrophy and dysfunction at late stage, whereas the early up-regulations of GDNF and NT-3 in EOMs are closely associated with the relatively well-preserved muscle morphology at late stage. Collectively, the data suggested that comparing NTFs expression between limb muscles and EOMs from different stages of ALS animal models is a useful method in revealing the patho-physiology and progression of ALS, and eventually rescuing motor neuron in ALS patients.

## Introduction

Amyotrophic lateral sclerosis (ALS) is an adult onset neurodegenerative syndrome. It is characterized by selective loss of both upper and lower motor neurons and progressive muscle wasting, paralysis and eventually death due to respiratory failure [1]. The incidence of ALS is highest between the ages of 50 to 70 years but it is also encountered before 40 years of age [2]. ALS occurs in a sporadic form (sALS) and a familial form (fALS). sALS occurs randomly with no strongly associated risk factors whereas fALS can be due to mutations in several genes, one of the most common genes being the gene coding for copper-zinc superoxide dismutase type 1 (SOD1). There are presently 177 known mutations in SOD1, which are related to the pathogenesis of ALS [3], [4]. Currently, SOD1^G93A^ mutant mouse model is the most widely studied ALS model because its unique and well-characterized feature in development of ALS-like symptoms [5].

Conventionally, it has been hypothesized that motor neuron death is the initial event in ALS, followed by muscle dystrophy and weakness. Recently, several researches have, however, demonstrated the importance of muscle in ALS pathogenesis. Dobrowolny et al. [6], [7] have shown that muscles with restricted SOD1 expression was sufficient to induce muscle atrophy and oxidative stress, thus muscle atrophy was independent on motor neuron degeneration in SOD1^G93A^ mice. It has also been shown that neuromuscular junctions in muscle tissue were involved early in the pathogenesis of ALS, as evidenced by dramatic denervation at neuromuscular junctions as early as around 50 days, before any loss of motor neurons in ventral horns [8]. Similar observations have been reported in other studies where neuromuscular junctions in limb muscles were affected before lower motor neurons were lost in spinal cord [9]–[11]. These findings suggest that alterations in muscles occur earlier than the presence of motor neuron degeneration in ALS.

Neurotrophic factors (NTFs) are a group of endogenous signalling proteins produced in different cells. Target-derived NTFs can be transported to lower motor neurons through different pathways: retrograde transport from muscle cells, anterograde transport from upper motor neurons, paracrine support from neighboring cells, i.e. microglia and Schwann cells, endocrine support from ependymal cells in the periphery, and autocrine support from the lower motor neuron itself [12]. NTFs can be classified into three subgroups: 1) the neurotrophin family which includes nerve growth factor (NGF), brain-derived neurotrophic factor (BDNF), neurotrophin-3 (NT-3), neurotrophin-4/5 (NT-4) and neurotrophin-6; 2) Glial cell line-derived neurotrophic factor (GDNF); and 3) neuropoietic cytokines.

NTFs promote survival and maintenance of specific populations of neurons and regulate neuronal differentiation [12], [13]. NGF was the first NTF discovered in salivary glands and it promoted mainly the growth of sensory and sympathetic nerve cells [14], [15]. Since NGF is limited in functions of regulating the development and survival of motor neuromuscular system, it was generally not considered as a true motor neuron trophic factor [16], [12]. BDNF has been proposed to promote motor neuron survival and motor axons growth [17]–[19] whereas GDNF has been shown to be able to rescue developing and adult motor neurons from programmed and injury-induced cell death [20]. NT-3 has been proved to be important in regulating muscle sensory neuron growth and maintaining proprioceptive sensory organs [21], [22] whereas NT-4 was revealed to be involved in growth and remodeling of adult motor neuron innervation [23], [24].

NTF dysfunction has been suggested to be associated with the pathogenesis of ALS: 1) mutation in some NTFs causes motor neuron disease; 2) expression of several target-derived NTFs is reduced in ALS; 3) the signaling pathways of some target-derived NTF are blocked in ALS [25]. It has been hypothesized that failure in secreting sufficient amounts of NTFs in muscles, glial cells and Schwann cells could lead to lower motor neuron degeneration, thus, loss of specific neuron population. As a consequence, that would in turn deprive the upper motor neurons of NTF support [12]. BDNF has been shown to protect neurons from *in vivo* excitotoxicity [26], a mode of action of relevance to ALS. Previous study using exogenous BDNF has successfully delayed motor neuron degeneration, reduced muscle atrophy and motor axon loss in wobbler mutant mice, an ALS animal model [27]. Similar effects have also been observed in another ALS mouse model (SOD1^G93A^) where exogenous GDNF delayed ALS onset, rescued motor neurons and increase lifespan [28], [29]. In addition, protective effects have been observed in NT-3 and NT-4 on motor neurons of ALS mouse model [25], [30]. Among the NTFs, BDNF has been considered as one of the most promising NTF in application of ALS clinical treatment. However, so far no clinical trial has been successful [25], [31].

The extraocular muscle (EOM) is fundamentally distinct from other skeletal muscles and classified as a separate muscle allotype [32], [33]. The uniqueness of EOM is demonstrated by its selective involvement in a number of diseases such as Myasthenia gravis, thyroid-associated orbitopathy and Miller Fisher syndrome [34]–[36], as well as by its selective resistance to a number of diseases such as Duchenne muscular dystrophy and merosin-deficient muscular dystrophy [34], [35], [37], [38]. We have recently demonstrated the uniqueness of EOM in ALS disease where the general cytoarchitecture were relatively well-preserved in comparison with limb muscles from late stage ALS patients [39]–[41].

In the present study, the mRNA levels of four different NTFs (BDNF, NT-3, NT-4 and GDNF) were examined in limb muscles and EOMs from ALS mice at early (∼50 days) and terminal stages (∼150 days), and from age-matched control mice. We aimed to investigate 1) the differences in expression of the NTFs in EOMs and limb muscles between the transgenic mice and the controls; 2) the differences in expression of the NTFs between EOMs and limb muscles in the transgenic mice. As NTFs are secreted by target tissues, it is crucial to understand the potential source fluctuations of NTFs that are altered in the process of ALS. Thus, we also aimed to investigate 3) the differences in expression of the NTFs in EOMs and limb muscles between different ALS stages.

## Materials and Methods

### Ethics statements

The animal study has been conducted according to national and international guidelines. Experiments and animal handling were approved by the Umeå ethical committee and were carried out in accordance with the European Communities' Council Directive (86/609/EEC).

### Mice

SOD1^G93A^ transgenic mice were originally obtained from Jackson Laboratories and were backcrossed with C57/BL6 Bom Tac for at least 20 generations (Bar Harbour, Marine, USA). Six SOD1^G93A^ mice at early stage around 50 days and six SOD1^G93A^ mice at terminal stage around 150 days were sacrificed for muscle sample collection. Age-matched healthy littermates served as early stage controls (∼50 days) and late stage controls (∼150 days).

### Muscle samples

EOMs were dissected from eyeballs whereas soleus and gastrocnemius muscles as a whole were dissected from hind limbs. Care was taken to excise the muscles in their extremity and free from other tissues. Muscle samples for histochemistry were mounted on cardboard and rapidly frozen in propane chilled with liquid nitrogen, and stored at −80°C until sectioned. Tissue samples collected for RNA analyses were submerged in RNAlater solution (Life Technologies Europe BV, Stockholm, Sweden) and incubated at +4°C overnight whereafter they were transferred and stored at −80°C until analysed.

### Histochemistry

Frozen specimens were sectioned in 7 µm thickness using a Reichert Jung cryostat (Leica; Nuss-loch, Germany) at −25°C. Sections were then stained for hematoxylin and eosin [42] for general morphological evaluation.

### RNA isolation and cDNA synthesis

Total RNA was extracted from muscle tissues using TRIzol Reagent according to the manufacturer's protocol (Invitrogen, Carlsbad, California, USA). RNA concentrations were determined using a ND-1000 spectrophotometer (NanoDrop Technologies, Wilmington, DE, USA) and integrity was assessed using an Agilent 2100 bioanalyzer and RNA 6000 Nano gel kit (Agilent Technologies, Waldbronn, Germany). RNA was stored at −80°C until use.

Total RNA (1 µg from limb muscles or 0.1 µg from EOMs) was reverse transcribed using random hexamers and TaqMan reverse transcription reagents (Applied Biosystems, Foster City, CA, USA). cDNA was stored at −20°C until further processing.

### mRNA quantification

Quantitative RT-PCR (qRT-PCR) was used to determine relative mRNA levels and was performed on an ABI Prism 7000 Sequence Detection System (Applied Biosystems) using TaqMan Universal PCR Master Mix and the following TaqMan gene expression assays (Applied Biosystems) containing predesigned primers and probes of the target, i.e. BDNF (Mm01334042_m1), GDNF (Mm00599849_m1), NT3 (Mm01182924_m1) and NT4/5 (Mm01701591_m1). All samples were run in triplicates according to the manufacturer's instructions. Gene expression was quantified using the relative standard curve method. To prepare appropriate standard curves for each gene, RNA was isolated and reverse transcribed as described above from tissues expressing relatively high levels of the target gene(s), i.e.; spleen (standard for NT3 and NT4), kidney (standard for BDNF) and cerebellum (standard for GDNF), respectively. Expression levels of target genes were normalized to β-actin using the Mouse ACTB (actin, beta) Endogenous Control assay (NM_007393.1; Applied Biosystems).

### Statistical analyses

All data are expressed as mean ± SD. Statistical analyses of comparisons were performed using one-way ANOVA/unpaired t-test (Statview 4.5; Abacus concepts, Inc., Berkeley California). *p*≤0.05 was considered to be statistically significant.

## Results

### Morphological alterations in EOMs and limb muscles over time

**Figure 1:**
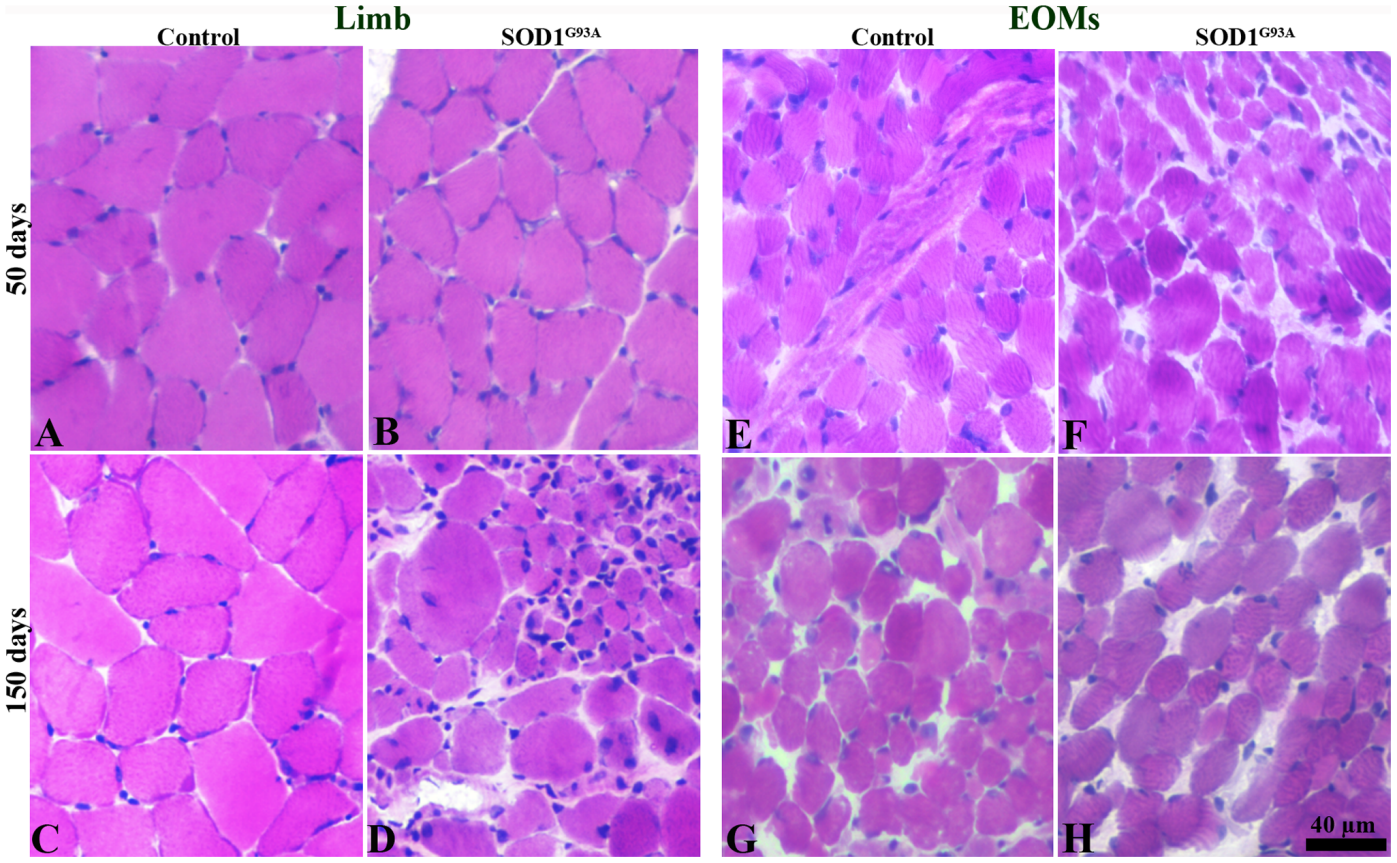
H&E staining of limb muscles (A–D) and EOMs (E–H) from control and SOD1^G93A^ mice at 50 days and 150 days. Normal morphological features of muscle fibres in limb muscles (B) and EOMs (F) at 50 days and in EOMs (H) at 150 days. In limb muscles of terminal stage SOD1^G93A^ mice (D), muscle fibres presenting pathological alterations including atrophy, hypertrophy, round fibres, and fibres with central nuclei.

In limb muscles of control mice at both ages, muscle fibres were in polygonal shape and tightly packed with peripherally located nuclei (Figure. 1A, C). In limb muscles of early transgenic mice, muscle fibres showed similar morphological features as in limb muscles of age-matched controls (Figure. 1B). However, small fibres were occasionally observed in the muscles of early stage transgenic mice. The results indicated that early stage of ALS has no significant impact on limb muscle morphology. In limb muscles of late stage transgenic mice, alterations in muscle morphology of both atrophied and hypertrophied fibres were frequently observed (Figure. 1D). Normal EOM muscle fibres were smaller, round and loosely arranged in muscle fascicles in mice of both ages (Figure. 1E, G). In contrast to limb muscles, muscle morphological features in EOMs in late stage transgenic mice were comparable to that of EOMs in late stage control mice (Figure. 1F, H).

### NTFs expression in limb muscles and EOMs of control mice

**Figure 2:**
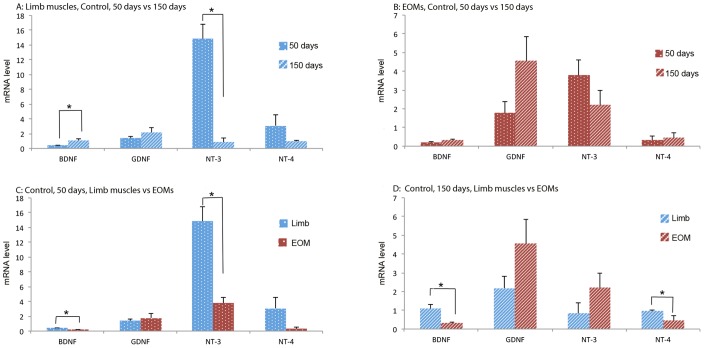
mRNA levels of NTFs in limb muscles and EOMs from 50 days and 150 days control mice. Top panels: comparison of NTFs expression in limb muscles (A) and EOMs (B) between 50 days and 150 days. Bottom panels: comparison of NTF expression between limb muscles and EOMs of 50 days (C) and 150 days (D). The expression levels are given using arbitrary units normalized by β-actin expression. Bars represent mean ± SD from analysis of at least 5 separate samples run in triplicate. *: *p*<0.05.

In limb muscles of the control group, mRNA levels were significantly increased in BDNF, but decreased in NT-3 over time (Figure. 2A). In EOMs of the control group, however, none of the NTFs was significantly changed in expression over time (Figure. 2B). Comparison of NTF expression between limb muscles and EOMs in early stage control mice revealed that mRNA levels of BDNF and NT-3 were significantly higher in limb muscles than in EOMs (Figure. 2C), whereas in the late stage control mice, expressions of BDNF and NT-4 were significantly higher in limb muscle than in EOMs (Figure. 2D).

### NTF expression in limb muscles and EOMs of ALS transgenic mice

**Figure 3:**
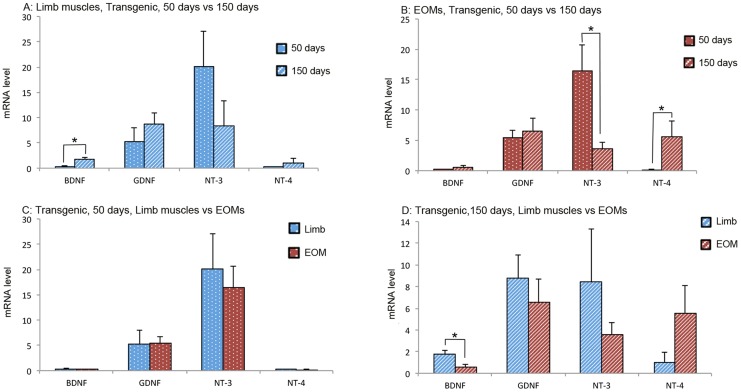
mRNA levels of NTFs in limb muscles and EOMs from early and late stage ALS mice. Top panels: comparison of NTF expression in limb muscles (A) and EOMs (B) between early and late stages. Bottom panels: comparison of NTF expression between limb muscles and EOMs of early (C) and late stages (D). The expression levels are given using arbitrary units normalized by β-actin expression. Bars represent mean ± SD from analysis of at least 5 separate samples run in triplicate. *: *p*<0.05.

In limb muscles of the transgenic mice, expression of BDNF was significantly increased over time (Figure. 3A). In EOMs of the transgenic mice, expression of NT-3 was significantly decreased whilst NT-4 was significantly increased over time (Figure. 3B). Comparison of NTF expression between limb muscles and EOMs in early stage transgenic mice revealed no significant difference in any NTFs (Figure. 3C). In late stage transgenic mice, only BDNF was significantly higher in limb muscles than in EOMs (Figure. 3D).

### Comparison between control and ALS transgenic mice

**Figure 4:**
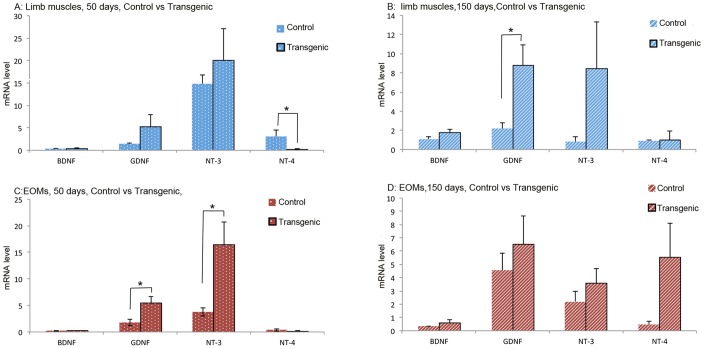
mRNA levels of NTFs in limb muscles and EOMs from early and late stages ALS mice and from age-matched control mice. Top panels: comparison of NTF expression in limb muscles between ALS mice and control mice of 50 days (A) and 150 days of age (B). Bottom panels: comparison of NTF expression in EOMs between ALS mice and control mice of 50 days of age (C) and 150 days (D). The expression levels are given using arbitrary units normalized by β-actin expression. Bars represent mean ± SD from analysis of at least 5 separate samples run in triplicate. *: *p*<0.05.

In limb muscles of early stage control and transgenic mice, mRNA level of NT-4 was significantly higher in controls than in ALS mice (Figure. 4A). However, in limb muscles of late stages control and transgenic mice, GDNF was significantly higher in transgenic mice than in control mice (Figure. 4B). In EOMs of early stage control and transgenic mice, expressions of GDNF and NT-3 were significantly higher in transgenic mice than in control mice (Figure. 4C). In EOMs of late stage control and transgenic mice, however, no difference was observed in any of the NTFs (Figure. 4D).

## Discussion

The present study for the first time examined NTF expression in different muscle tissues from different ages of both transgenic and control mice. The results showed that in early stage ALS mice, GDNF and NT-3 were significantly up-regulated in EOMs whereas NT-4 was markedly down-regulated in limb muscles. Taken the morphological observations into consideration, we believe that the early alterations in NTF expression were closely associated with the progression of ALS.

### BDNF

Previous observations in BDNF expression in skeletal muscle tissue are controversial. Some studies have failed to detect BDNF in muscle tissues of adult mice and rats by either in situ hybridization [43] or Western blotting [44], whereas others have observed very low levels of mRNA and protein in skeletal muscles of both young and adult mammals [17], [45]–[47]. During embryonic development, absent and low [48] as well as high mRNA levels [49] of BDNF have been observed in muscle tissue, followed by slight up-regulation in adult muscle tissue [48]. The current results showed that mRNA level of BDNF was not significantly affected in either muscle tissue in either early or late stage transgenic mice, suggesting that BDNF expression in both limb muscles and EOMs was not associated with ALS. This is consistent with previous studies on ALS patients where mRNA expression of BDNF was not significantly affected in muscle tissues [50].

BDNF has been demonstrated to promote motor neuron survival and motor axon growth [17]–[19], therefore has been studied in a variety of neurodegenerative conditions, including Parkinson's disease and ALS [51], [52]. Although pre-clinical trial of BDNF appeared promising, the clinical trial failed [53]. On basis of the present results that BDNF was not closely associated with ALS, BDNF seems not to be a potent candidate in motor neuron rescuing.

### GDNF

Up-regulation of GDNF expression has been reported in previous studies on both ALS mouse models and on ALS patients. Up-regulation of GDNF was suggested to be associated with motor neuron supporting and rescuing [50], [54]. Saarma & Sariola [55] showed that GDNF had a 100-fold higher efficacy in rescuing spinal motor neurons as compared to other NTFs including BDNF, NT-3 and NT-4. Overexpression of GDNF in gastrocnemius muscle has been shown to present hyper-innervation at neuromuscular junctions in transgenic mice beyond the normal development period [56]. Treatment of ALS mice with GDNF prevented motor neurons from degeneration, preserved axon innervation and inhibited muscle atrophy [57].

In the present study, GDNF was significantly up-regulated in EOMs of early stage transgenic mice. Interestingly, in this study and our previous studies on terminal ALS mice and patients, we observed that EOMs maintained relatively well-preserved cytoarchitecture, intact innervation of motor endplates and normal expression of laminins, synaptophysin, and p75 neurotrophin receptor at neuromuscular junctions [19], [20], [55]. We hypothesize that the up-regulation of GDNF in EOMs at early stage is triggered by the early development of ALS, leading to protection of EOMs from degeneration, and thus resulting in the well-preserved EOM morphology.

In contrast, in limb muscles of ALS mice, GDNF was significantly up-regulated at terminal stage but not at early stage. Coincidently, the limb muscles at terminal stage were in severe denervation and had an abnormal distribution of laminins, synaptophysin, and S-100 at synapses [39], [40], [58]. Therefore, the up-regulation of GDNF in limb muscles seems to be a secondary effect of motor neuron degeneration [59]. However, if taking the results of both EOMs and limb muscles into consideration, GDNF seems to play a role in protecting motor neuron from degeneration. The scenario is that the development of ALS initiates an early up-regulation of GDNF in EOMs, resulting in well-protected EOMs. However, ALS did not trigger an early up-regulation of GDNF in limb muscles, therefore resulted in muscle degeneration in the late transgenic mice. The high level of GDNF in limb muscles of late stage transgenic mice may be an accumulation due to failure of retrograde transportation caused by denervation or may be associated with motor neuron rescuing, but in vain.

### NT-3

NT-3 has been proposed to be important in regulating muscle sensory neuron growth and maintaining proprioceptive sensory organs [21], [22]. Lacking of NT-3 led to deficiencies in peripheral nervous system, and loss of limb proprioceptive afferents, whereas over-expression of NT-3 resulted in enhanced survival of proprioceptive sensory components [60], [61]. NT-3 has been shown to be abundant during embryonic development and in postnatal phase [49], and high level of NT-3 in skeletal muscles seemed to be maintained throughout life [47]. According to previous studies, we hypothesized that NT-3 would not be significantly changed following the time course. However, in the present study, NT-3 was significantly down-regulated in limb muscles of the control mice from 50 days to 150 days. Presently, we were unable to explain the controversial results, and perhaps more tissue sampling time-points would help to reveal the truth.

Diverse alterations of NT-3 in muscle tissues and spinal motor neurons have been observed in ALS patients. In several studies on ALS patients, NT-3 has been reported to be up-regulated in biceps brachii muscle [59], but down-regulated in spinal motor neurons [62]–[64]. It was believed that the motor neuron pathology in ALS triggered up-regulation of NT-3 in muscles and NT-3 was then transferred retrogradely via axonal transport to spinal cord to rescue the motor neurons [17], [18], [65], [66].

In the present study, NT-3 in EOMs was significantly higher in early transgenic than in early control mice. Similar to GDNF, the early up-regulation of NT-3 in EOMs may also be triggered by early development of ALS to compromise the early impact of ALS on EOMs, leading to the relatively well-preserved EOM morphology. On the contrary, insufficient up-regulation of NT-3, as observed in the limb muscles of the study would fail to rescue the motor endplates and motor neurons, leading to motor endplate denervation [8], [58] and resulted in the present observation of muscle degeneration.

### NT-4

Previous studies have shown that the expression of NT-4 was ubiquitous and less affected by environmental signals compared to other NTFs [67], [68]. Mice lacking NT-4 exhibited enlarged fragmented neuromuscular junctions with disassembled postsynaptic acetylcholine receptor clusters, reduced acetylcholine receptor binding, and acetylcholinesterase activity [68]. Thus, NT-4 was proposed to be involved in growth and remodeling of adult motor neuron innervation by means of coordinated adaptation of neuromuscular performance to electronic stimulation and muscle exercise [23], [24]. NT-4 was not extensively studied in ALS patients. In a recent study on ALS patients, mRNA level of NT-4 was significantly up-regulated in muscle tissue and the up-regulation was amplified following the development of ALS [59].

In the present study, NT-4 in limb muscles was significantly down-regulated in early ALS mice compared to age-matched controls (Figure. 4A). Following the time course of ALS, NT-4 in limb muscles was, however, not affected further (Figure. 4B). On the contrary, NT-4 level in EOMs was not significantly different between transgenic and control mice at either stage (Figure. 4C, D), but was indeed significantly up-regulated following the time course of ALS (Figure. 3B). The results indicated that the early stage of ALS was associated with early down-regulation of NT-4 in limb muscles, whereas late stage of ALS was associated with significant up-regulation of NT-4 in EOMs.

Previous study on SOD1^G93A^ mice has shown that denervation of neuromuscular junction occurred at early symptomatic stage, long before motor neuron loss in spinal cord [8]–[10]. We proposed that the early down-regulation of NT-4 in limb muscles might be associated with early retraction of axons from neuromuscular junctions [8], [9], leading to the late stage limb muscle degeneration. Indeed, previous study has detected an impaired neuromuscular transmission as evidenced by significant decrease in amplitude of both endplate potentials and miniature endplate potentials in SOD1^G93A^ mice at early symptomatic stage [69]. Taken together, we concluded that in ALS mice, early decrease in NT-4 expression in skeletal muscles is closely associated with late stage ALS mice of muscle dystrophy and dysfunction.

Contrary to limb muscles, in EOMs of early stage ALS mice, GDNF and NT-3 were significantly up-regulated. This was believed to provide sufficient trophic support to peripheral neuronal axons in EOMs at early stage of ALS, leading to intact neuromuscular junctions, and resulted in the present finding of well-preserved EOMs at late stage of ALS. These speculations were also supported by our previous findings that EOMs were less affected than limb muscles in late stage ALS mice and ALS patients [39], [41], [58], and that neuromuscular junction was intact in EOMs but was denervated in limb muscles at late stage of transgenic mice [40], [58].

ALS is an adult-onset neurodegenerative disease. However, the present study showed that alteration in NTF expression occurred early when no sign of denervation of motor neuron or muscle dysfunction appeared. Thus, a thorough understanding of NTFs' expression patterns in limb muscle and EOM is very important in revealing the pathophysiology and progression of ALS, and eventually rescuing motor neuron in ALS patients.
